# Supplementary material for: Seminoma and Embryonal Carcinoma Footprints Identified by Analysis of Integrated Genome-Wide Epigenetic and Expression Profiles of Germ Cell Cancer Cell Lines
Source: PLoS One. 2014 Jun 2;9(6):e98330. doi: 10.1371/journal.pone.0098330 (PMC4041891; doi:10.1371/journal.pone.0098330)
Supplement: File S1 — ZIP file containing DMRforPairs output for significant regions. Please start from the html files. (ZIP) [file pone.0098330.s008.zip › figures/41587.pdf]

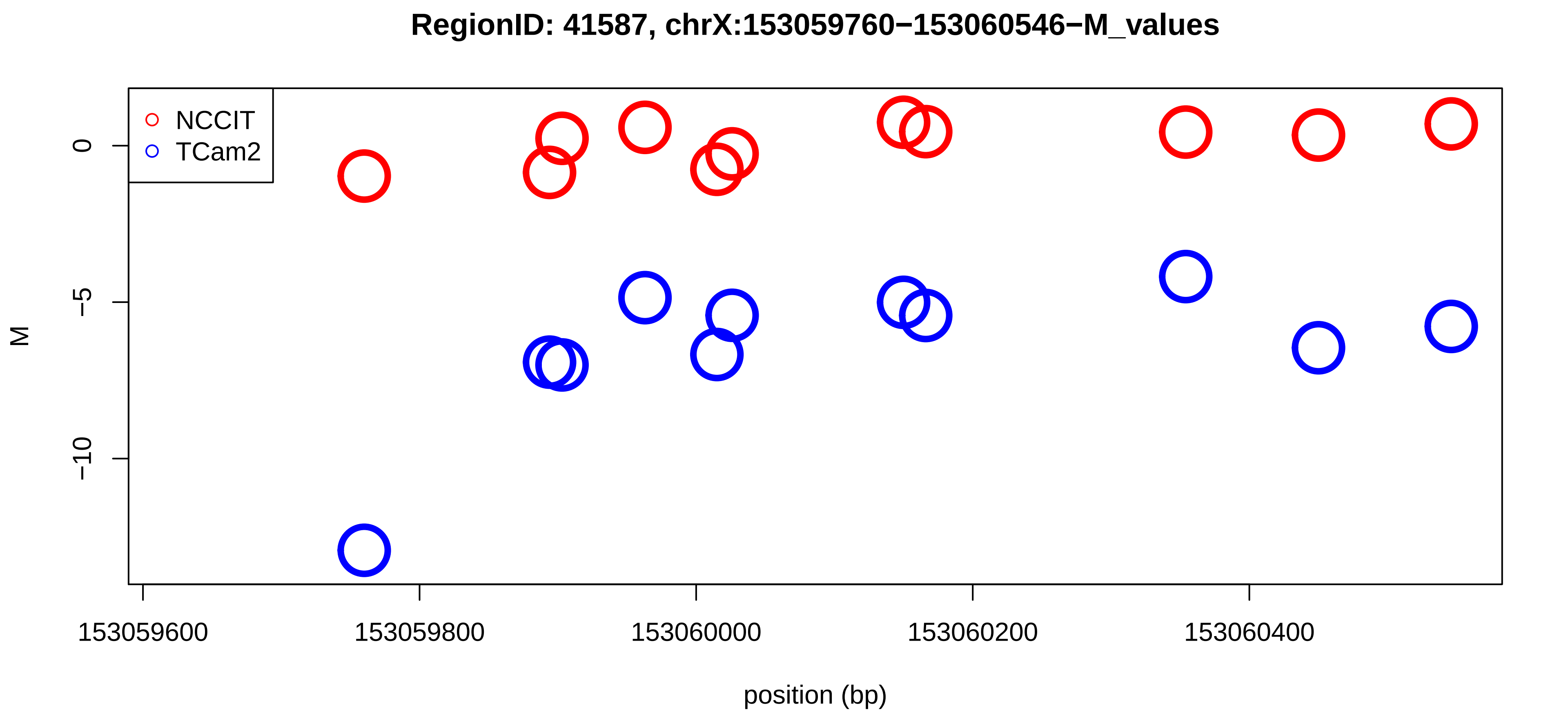
RegionID: 41587, chrX:153059760−153060546−M_values
NCCIT
TCam2
M
0
−5
−10
153059600
153059800
153060000
153060200
153060400
position (bp)

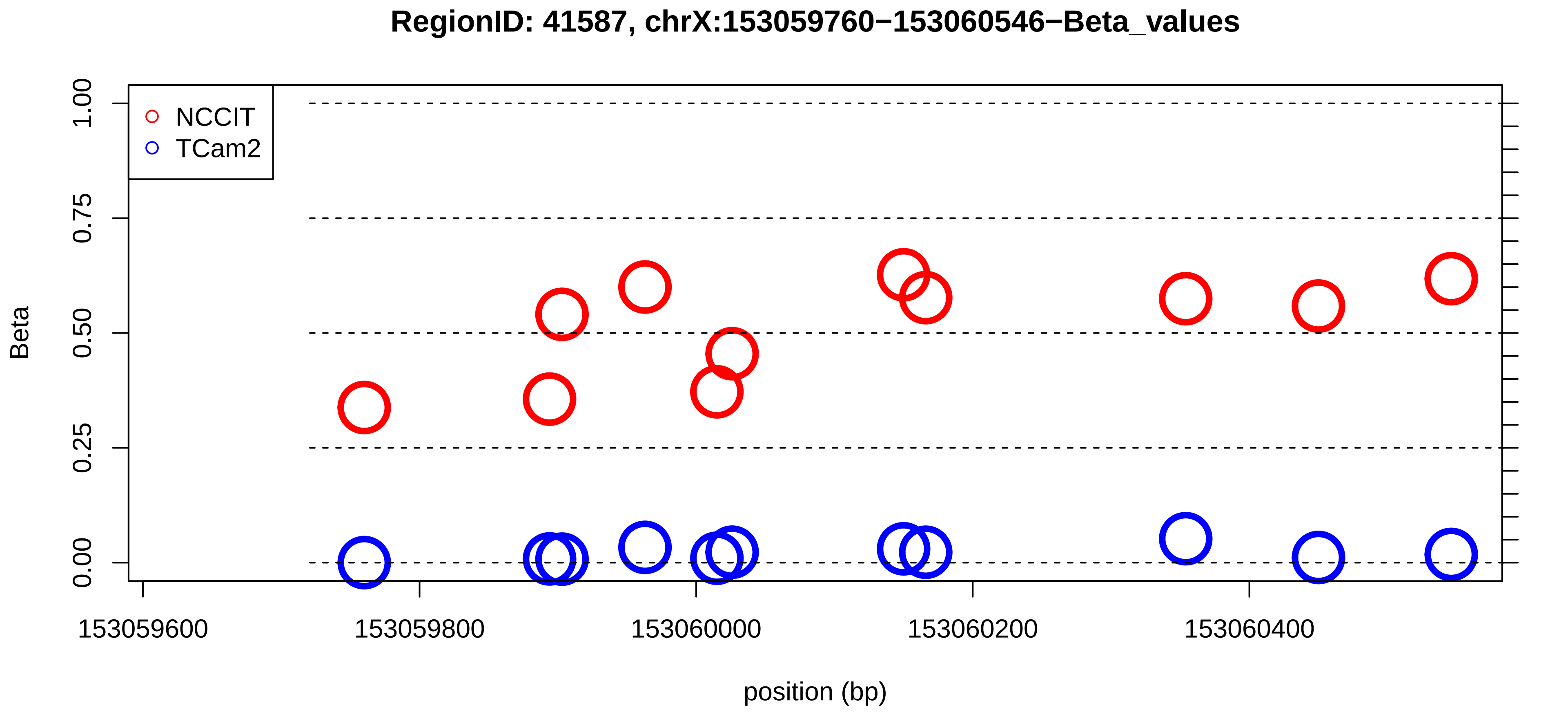
RegionID: 41587, chrX:153059760−153060546−Beta_values
NCCIT
TCam2
Beta
0.00
0.25
0.50
0.75
1.00
153059600
153059800
153060000
153060200
153060400
position (bp)

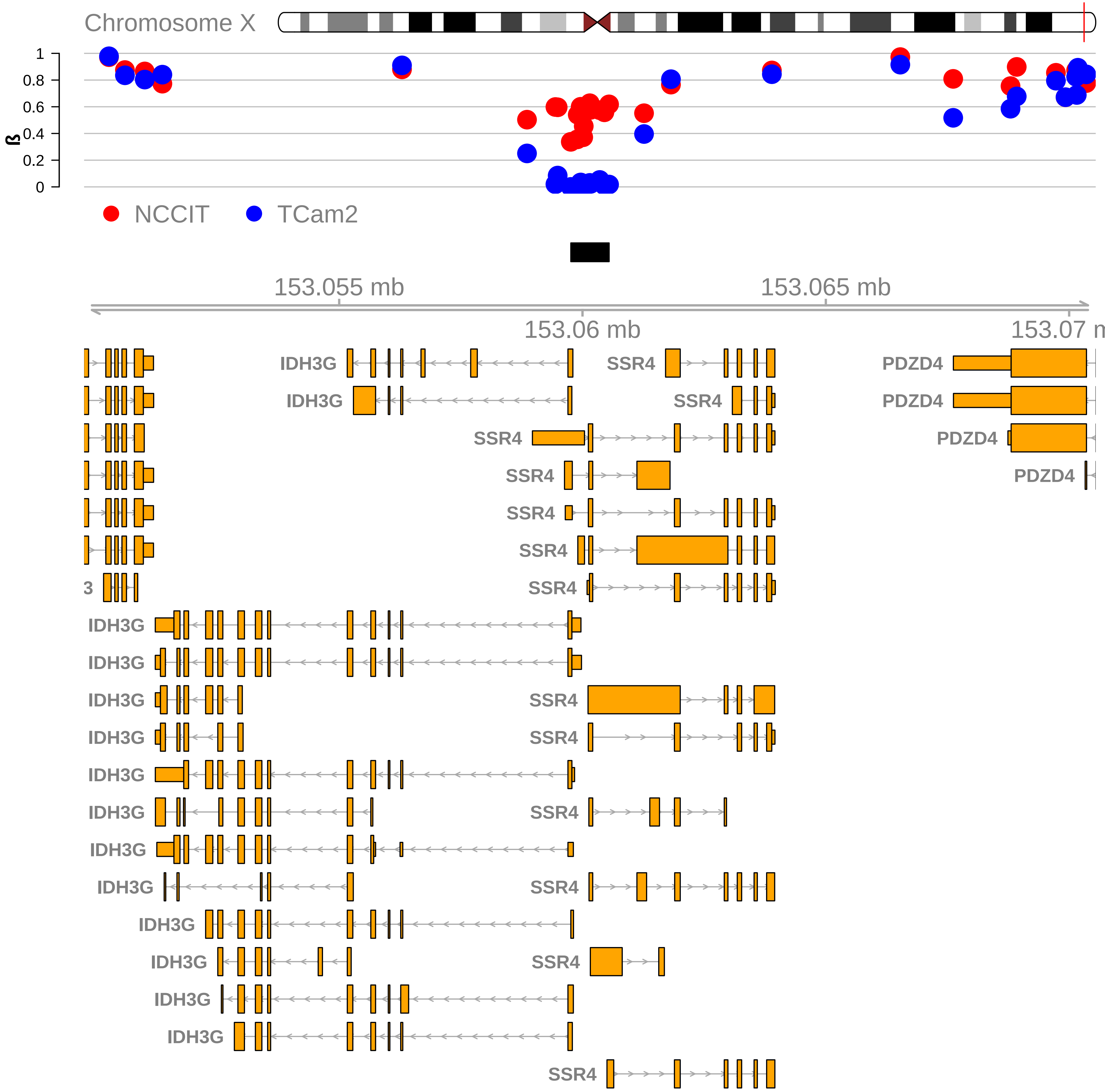
Chromosome X
ß
1
0.8
0.6
0.4
0.2
0
NCCIT
TCam2
153.055 mb
153.065 mb
153.06 mb
153.07 m
IDH3G
SSR4
PDZD4
